# CORE: Compassion Oriented Reflection and Engagement to Guide Academic-Community Partnership

**DOI:** 10.35844/001c.13314

**Published:** 2020-07-21

**Authors:** Tommy Chou, Stacy L. Frazier

**Affiliations:** 1 Psychology, Florida International University (FL)

**Keywords:** community-based organizations, compassion, academic-community partnership, poverty

## Abstract

Estimates in dissemination, implementation, and services (DIS) research continue to present a 17-year lag for implementation of only 14% of evidence-based clinical services and technologies in practice (Chambers, 2018) - especially troubling for communities characterized by disproportionately high rates of poverty, crime and mental health need (Yoshikawa, Aber, & Beardslee, 2012). Academic-community partnerships offer pathways by which to speed the transport of evidence-based innovations; however, a range of challenges can disrupt implementation and adoption (Damschroder et al., 2009). This manuscript presents Compassion-Oriented Reflection and Engagement (CORE), a framework to inform academic collaborators’ perspectives and practices towards building flexible, responsive partnerships with youth-serving community-based organizations.

Recent decades have seen a rapid expansion in dissemination, implementation, and services (DIS) efforts in healthcare; however, current literature continues to estimate a 17-year lag for implementation, and only 14 percent overall adoption of evidence-based clinical tools and technologies in practice (see Chambers, 2018 for a review). While investigators know less about rates of penetration for science specific to families impacted by resource scarcity, a robust body of work documents barriers to crucial evidence-based practices (EBP) in communities characterized by high rates of poverty (Yoshikawa et al., 2012). Youth in economically disadvantaged urban city centers continue to attend underfunded, understaffed schools (Cappella et al., 2008), face higher rates of domestic and community violence exposure (Foster & Brooks-Gunn, 2009), and contend with geographic barriers (Baker et al., 2006) that altogether interfere with meeting basic physical, educational, and mental health needs.

To address this gap, DIS scientists have increasingly leveraged a wide variety of academic-community partnerships (ACP) to transport EBPs to socioeconomically vulnerable communities (e.g., Fagan et al., 2012). Systematic cultivation of ACPs has yielded a robust body of work describing conceptual models to maximize the success of EBP implementation in community-based organizations (CBOs) (see Consolidated Framework for Implementation Research, Damschroder et al., 2009), and investigators have presented frontline challenges and examples of problem solving, “lessons learned,” and recommendations for the procedures of ACP (Frazier et al., 2019; Stetler et al., 2006). In contrast to the well-established literature describing *what to do* (e.g., specific steps) in partnership, a growing discourse has increasingly called for guidance in the process of ACP, or *how to do it* (e.g., ethics, Campbell & Morris, 2017; Chou & Frazier, 2019). To this end, we introduce Compassion-Oriented Reflection and Engagement (CORE), a process model guiding our role and function as academic partners and describe its development and application through our experience as community-engaged researchers working in collaboration with a youth-serving non-profit organization called *Champions* (a pseudonym).

## Compassion-Oriented Reflection And Engagement

Compassion-based theory serves as a fitting springboard from which to consider the process of partnership for a number of reasons. First, prior research presents compassion-based practice as trainable and beneficial in guiding *process* (e.g., Beaumont & Hollins Martin, 2016; Sinclair et al., 2017), as well as *procedure* (evidence- and compassion-based interventions have shown promise in implementation and knowledge translation, Sapthiang et al., 2019). Therefore, training in compassion-based practice may prove especially efficient and effective for community-engaged scientists. Second, compassion-based strategies align well with current ACP procedural models promoting open engagement with community partners, joint decision-making about collaborative goals, and a mindful regard and consideration for power dynamics (e.g., as in Community-Based Participatory Research [Belone et al., 2016] and the Consolidated Framework for Implementation Research [Damschroder et al., 2009]). Third, studies in experimental psychology, as well as discourse in healthcare and education, point to compassion-based practices such as meditation and mindfulness as routes by which to reduce implicit bias toward marginalized groups in practice (Burgess et al., 2017; Carson & Johnston, 2000; Kang et al., 2014) - a phenomenon that may prove indispensable in healing damaged trust and regard among historically disenfranchised groups toward scientists and providers. Lastly, emerging evidence points to *self*-compassion as a means to bolster resilience and ameliorate the effects of burnout and vicarious trauma (Knight, 2013; Scarlet et al., 2017). As such, a practice built on compassion may prove protective for both academics and community stakeholders, individually and in partnership.

Compassion-Oriented Reflection and Engagement - like many psychotherapeutic tools with a compassion focus (e.g., mindfulness, meditation) - draws heavily on traditional Buddhist theory and principles (Shonin et al., 2014). Compassion is a multi-faceted construct defined in a variety of ways over many centuries. Brill & Nahmani (2017) draw on a number of sources - including the Dalai Lama, a preeminent figure in Buddhist and Eastern philosophies (Shonin et al., 2014) - and consolidate various conceptualizations to describe compassion in three components: acknowledgement of others’ suffering, empathy for their experienced pain, and action to relieve suffering. To be clear, “suffering” here refers broadly to challenging experiences and related distress, and “compassion” requires recognizing the sources and outcomes of distress; perspective taking and responding to the distress; and acting to help alleviate it, for instance by removing a source of pain, offering tools for healing, or improving capacity for coping. Fundamentally, CORE guides community-engaged researchers’ practices to build rich, genuine relationships with local partners by encouraging them to attend mindfully and non-judgmentally to stakeholders’ perspectives, context, and goals, and employing flexible thinking to arrive at joint solutions.

## Learning Together With Our Community Partner: *Champions*

*Champions* is a non-profit organization working to build the capacity of children and families. It employs a block-by-block model to support local neighborhoods with high rates of poverty and violent crime (The Metropolitan Center, 2016). Families there predominantly identify as Black/African American, and local history documents a long narrative of disenfranchisement and injustice. *Champions* provides a range of health, education, and employment services (e.g., transportation, access to computers, resources for job searches and interviews, health and wellness initiatives, and a fresh food co-op), including afterschool and summer programming for preschool, elementary, middle school, and high school age youth, held at nearby public schools.

### *Champions* Organizational Hierarchy

*Champions* operates out of a main office and multiple sites in the neighborhood including community offices in residential complexes and schools hosting afterschool and summer programs. Specifically, they invited our collaboration to support their afterschool and summer programming, for children enrolled in preschool through 8^th^ grade, held at a local K-8 magnet school that also houses a Head Start program. We engaged across levels of the organizational hierarchy - with leadership, site supervisors, and frontline staff.

#### Leadership

Leadership included *Champions*’ CEO (n=1) and program directors (n=2–4) located predominantly at the *Champions* head office. Each member of leadership carries a range of responsibilities including grant writing, management of program budgets and payroll, selection of program curricula, and communicating both with each other and with site supervisors.

#### Site Supervisors

Site Supervisors provide on-site management, engage with children and families enrolled in afterschool and summer programming around logistics and major concerns (e.g., registration, field trips and events, absenteeism, disciplinary issues), and facilitate the exchange of information about program needs, resources, goals, and changes between frontline staff and leadership. Historically, one site supervisor presided over both pre-k and elementary (i.e., kindergarten through fifth grade) programs. During the last few years, *Champions* created a second site supervisor position for its new middle school program (i.e., grades 6 to 8).

#### Frontline Staff

Frontline Staff consisted of two groups: certified teachers (*n*=10–12) and “student supporters” (*n*=10–14). Certified teachers led supplemental lessons and provided homework support, while student supporters assisted in classroom management, supervised unstructured time (i.e., snack and recreational time), engaged students in outdoor games and activities, and communicated with students’ daytime schoolteachers to identify areas of growth. Frontline staff were frequently reassigned to different classrooms and programs, in part reflecting a revolving door of AmeriCorps members and volunteers, fluctuations in funding, partnerships with other local CBOs, and the implementation of “on call” or backup staff positions.

### ACP Goals And Activities

Though our community-engaged research team has collaborated with *Champions* for a number of years - typically as invited facilitators to staff workshops and trainings - we approached them in the fall of 2016 to establish a more systematic ACP defined by shared goals and equitable decision-making, with first author Chou acting as the primary academic partner. In our early meetings with *Champions*’ leadership, we converged around workforce support for frontline staff in *Champions*’ pre-kindergarten afterschool program as one of our primary partnership goals. Specifically, we scheduled three monthly meetings (“summits”) in the spring of 2017 to discuss socio-emotional learning and student engagement strategies accompanied by weekly site visits to observe, model, and consult on social-emotional content in real time. As we neared the end of our original ACP timeline, high-enthusiasm requests from frontline staff, leadership, and site supervisors for continued partnership led to our decision to extend collaboration. We established plans to revisit goals and activities at the start of each semester and came to a joint understanding that we would gradually transition to a less intensive model of partnership in the third year, coinciding with the first author’s timeline to complete graduate training.

Within the first year, monthly summits and weekly consultation expanded to incorporate the elementary (serving approximately 120–150 children each year) and middle school (serving approximately 30–50 children each year) programs, including direct support for both site supervisors and frontline teams. Together with stakeholders from all levels of *Champions*’ hierarchy, we shifted the focus of consultation to broader organizational strengths and barriers to quality programming in the second year. In addition to continuing our discussions on socio-emotional learning and student engagement, we incorporated topics such as communication, culture and climate, and staff burnout, responding in part to high turnover and frequent transitions in leadership as a number of *Champions* team members left the organization over the course of our three-year ACP (two from CEO positions, six from program director positions, three from site supervisor positions, and roughly ten from frontline staff positions). Of special note, though not explicitly part of our originally intended role and function, we invested significant time in supporting *Champions* and their community - at their request - through a number of tragic and sorrowful events including several related to gun violence, family conflict, and grief following the loss of students or staff. To a meaningful extent, broadening our role in this way revealed the significant and important contribution of compassion-based concepts and skills towards joining authentically and collaborating fully with the *Champions* team.

## Development And Application Of Core

In our efforts to provide flexible, responsive consultation, we sought to highlight and elevate the many strengths of the *Champions* team and engaged members at all levels of the workforce hierarchy to guide the activities and direction of our collaboration. We drew evidence-based practices from a range of psychosocial frameworks (e.g., cognitive behavioral therapy; Acceptance and Commitment Therapy; couples therapy), and employed strategies found in organizational and leadership interventions for youth service settings (e.g., Aarons et al., 2015; Glisson & Schoenwald, 2005). We increasingly found that the compassion-oriented literature resonated with our goals and process.

Over time, CORE began to frame our efforts and expectations as community-engaged researchers and to inform our practice, becoming part of our code of conduct alongside ethical guidelines and principles. In particular, CORE shaped our efforts as academic partners related to four themes: (1) thinking flexibly to build a responsive partnership; (2) promoting effective communication within the CBO; (3) responding to stress and emergent events impacting CBO staff; and (4) facilitating self-care and peer support within our research team. For each, we detail a relevant experience in ACP with *Champions*, discuss a compassion-based concept that supported our efforts to move forward in partnership with the *Champions* team, and consider its application to our work as well as its potential utility in other ACPs.

### Theme 1: Thinking Flexibly To Build A Responsive Partnership

Throughout our collaboration with *Champions*, we have continually expanded and redefined our role and objectives guided explicitly by the organization’s evolving goals and shifting priorities. When our collaboration began toward the end of 2016, we planned three monthly summits in early 2017, to be accompanied by weekly site visits for observation and in vivo support of EBP implementation. Summits were planned with leadership to include discussion and evaluation of staff norms, values, and perceived strengths and obstacles (January); empirically-supported content on emotions and mental health, student engagement, and strategies to address challenging behaviors (e.g., Good Behavior Game, differential attention, safe time-outs; February); and problem solving around implementation (March). Though partnered activities were initially intended for the preschool program alone, support was extended to the elementary program staff within the first month by request of the site supervisor. Additionally, the community team and high school program staff periodically joined monthly meetings but did not receive weekly consultation.

The expanded audience raised a much broader constellation of concerns than we previously planned to address. In addition to organizational challenges that included (but extended beyond) barriers relevant to implementation of recommended content, concerns included community building, parent engagement, mental health and trauma, supporting youth through family and community disruptions, and a host of others directly or indirectly reflecting resource scarcity. As a research team, our early conversations focused on providing support with sustainability in mind. We tried to minimize reliance on our consultation by prioritizing knowledge transfer and mobilization while leveraging, without overextending, local staff, structures, and resources. Organizational barriers (e.g., lack of structured activities, last-minute changes to daily routine, insufficient materials for instruction, limited control over classroom design and outdoor space), alongside staff turnover (including the CEO and program directors), limited the effectiveness of change efforts requiring minimal investigator support. Thus, we worked to reevaluate our role and function in partnership. To do so, we drew in part from Relational Frame Theory, a fundamental component of Acceptance and Commitment Therapy.

#### Compassion-Based Concepts

Relational Frame Theory states that individuals understand concepts largely in relation to other concepts, and these connections form semantic networks that ultimately drive their behaviors (Hayes et al., 2006). In Acceptance and Commitment Therapy, a therapist may apply this concept to help a patient understand their traumatic stress reaction to a car accident by explaining that their experience of a life-threatening event has created a connection with high salience between related stimuli (e.g., driving or riding in cars) and abstract concepts (e.g., danger, fear, risk of harm) (Hayes, 2018). In some cases, these semantic links - or relational frames - can become impairing (e.g., anxiety around driving prevents them from commuting to work) and stable enough to persist despite immediate, contradicting information, resulting in “cognitive fusion” (for more, see Hayes et al., 2006). Acceptance and Commitment Therapy embraces “cognitive defusion”, to help “loosen” rigid semantic ties, for instance through exposures, whereby patients gradually and safely engage with stimuli related to their anxiety (e.g., riding as a passenger on progressively longer car trips, ultimately driving to work again) to provide more *safe* experiences of driving and weakening the cognitive relationship between driving and danger.

#### Application

For more abstract problems, individuals can identify relational frames that may interfere with flexible and responsive collaboration and reappraise rigid connections through a mindful, nonjudgmental evaluation of thoughts and beliefs. This practice has allowed us to bring our time and effort more fully to the challenges most relevant to the *Champions* team. Toward addressing organizational barriers, we recommended strategies that minimized reliance on our team as the “middle man”; however, despite stakeholder enthusiasm, we found it difficult to maintain enough momentum to produce meaningful change given the numerous and competing demands impacting the *Champions* workforce. Evaluating our relational frames, we identified our own beliefs that *sustainable solutions are good*; and sustainable solutions in consultation require *minimal investigator support*, enabling them to remain viable after partnership ends. Thus, solutions that *require more than minimal investigator support* are not sustainable, and by extension, are *not good*. We ultimately recognized that *Champions* limited resources, time, and staff offered very few “degrees of freedom”; hence, our adherence to traditional definitions of sustainability and success from DIS science were impairing our ability to promote meaningful and lasting change. While we continue to appreciate the importance of conventions for science and practice, our early experiences with *Champions* compelled us to loosen boundaries defining our work and create flexibility to address pressing issues while maintaining those essential constructs that support ethical, rigorous science.

#### Lessons Learned

Processes and practices from Acceptance and Commitment Therapy supported our ability to contribute responsively to our partnership with *Champions*; specifically, we applied cognitive defusion in a series of steps to join their efforts at organizational change. First, we evaluated the guidelines and assumptions set forth within our research team that might restrict the options generated in our attempts at problem solving, both internally and with our partners. Second, we identified restrictive conditions that narrowed the possible mechanisms of change available in our collaboration (i.e., our conceptualization of and emphasis on sustainability). Lastly, we re-appraised the extent to which these pre-existing notions were applicable to the situation at hand, feasible given our goals and available resources, and truly necessary to progress. We revised our role to become more active participants in their organizational change process, leveraging our time and effort as added resource to create space for lasting growth (see Frazier et al., 2019).

### Theme 2: Promoting Effective Communication Within The Cbo

As we shifted our focus more intentionally and effortfully onto structural barriers impeding EBP adoption, we identified poor communication among frontline staff, site supervisors, and leadership as a chief concern across CBO levels. We utilized components of organizational and leadership interventions (Aarons et al., 2015; Glisson & Schoenwald, 2005) to assess and address workforce needs, extending consultation to site supervisors and program directors at their request. Specifically, we introduced conversations around communication and transformational leadership (Aarons et al., 2015), encouraged systematic collection of feedback from frontline staff through surveys and small group meetings, and ultimately proposed a stakeholder advisory structure informed by goals and principles of Organizational Action Teams (Glisson & James, 2002). Efforts to enhance and systematize communication across service, managerial, and executive levels revealed strained – and, in some cases, fractured – relationships across the organizational hierarchy: leadership worried that frontline staff would not be forthcoming in sharing concerns openly and honestly, while frontline staff doubted their efforts would be rewarded with meaningful change or follow through. At each level, there appeared to be doubts that other stakeholders were committed to promoting progress. In turn, partners expressed low overall expectations for the possibility to create meaningful improvements and, as a result, ambivalent engagement with the process.

Of particular interest, overlap in narratives told by frontline staff and leadership indicated convergence around (1) a joint mission to support local youth and families and (2) experiences of high workload and burnout. Conversations aligned with a well-documented bi-directional relationship between job stress and burnout, and interpersonal conflict (Ashill & Rod, 2011; De Dreu et al., 2004). On a larger scale, symbolic interactionism - which posits that individual communication and interpersonal processes of members within a social organization define the overall social environment (Maines, 1977) - suggests that this cycle of burnout and conflict likely fed into the overarching organizational culture and climate that, in turn, cycled back down to the workforce and affected burnout and readiness for change (Aarons et al., 2015; Glisson & James, 2002) Efforts to repair and restore positive, productive interpersonal dynamics, and to disrupt concentric cycles of burnout, conflict, culture, and climate led us to employ concepts from relationship/couples’ interventions to encourage development of safe and supportive working relationships. Couples’ therapy frameworks - which draw heavily on attachment theory - became a platform for partnered discussion built around the conceptual connection between compassion and secure attachment.

#### Compassion-Based Concepts

Mikulincer and Shaver (2005) propose that “if only people could feel safer and less threatened, they would have more psychological resources to devote to noticing and reacting favorably to other people’s suffering,” (p. 34) highlighting mutual compassionate regard as a potential pathway to achieving security in attachment by establishing that individuals will protect and support each other under circumstances of stress and hardship. In keeping with this perspective, we predominantly utilized speaker-listener strategies from the Prevention and Relationship Enhancement Program (Owen et al., 2012). Ultimately, individual and small group conversations moved through three stages: listening and validating concerns and perspectives through speaker-listener reflections; offering alternative explanations and generating empathy and compassion for peers; and problem solving.

#### Application

Accordingly, during consultation we adopted a coaching role resembling that of therapists facilitating speaker-listener exercises. In group discussion, for example, we often paused the conversation and asked site supervisors to reflect what they heard in concerns raised by their frontline staff, seeking confirmation or clarification from frontline staff as needed. Similarly, when program staff brought concerns to us individually, we reflected and validated their experience, and also provided alternative interpretations that might gently counter their assumptions. For example, when leadership attributed problems in program delivery to inadequate effort or devotion by frontline staff, we recognized their desire to see team members work proactively to provide high-quality academic support; at the same time, we pointed to routine challenges facing teachers and student supporters on a daily basis, coupled with scientific evidence about the impact of burnout on job performance. Conversely, when frontline staff interpreted poor communication as indicating indifference by program directors and site supervisors, we acknowledged their desire for increased oversight and support while detailing leadership’s numerous (often invisible) responsibilities (e.g., grant writing, fundraising, networking with local agencies, paperwork to document program activities) that may interfere with more direct engagement. We often leaned on an “iceberg” analogy, suggesting to partners at all levels that their perceptions of peers were based largely on a small, observable segment of others’ work - the “tip of the iceberg” - and often may not account for significant effort that occurs outside of their view.

#### Lessons Learned

Creating time and space for disclosing work that occurred “below the surface” to one another generated more widespread appreciation for the respective contributions by all *Champions* staff to support the community, which in turn facilitated cooperative problem solving. Importantly, strategies from couples’ therapy supported efforts towards encouraging effective communication. First, we used active listening skills - namely reflection (e.g., “What I hear you saying is…” followed by a brief summary and an opportunity for the other person to correct or clarify our understanding of their message) - to establish a working knowledge of each individual *Champions* staff members’ perspectives, goals, and lived experience. In doing so, we obtained a stronger foundation from which to facilitate conversations between individuals.

Second, we were careful to use the words “you think,” to highlight when individuals were expressing their subjective experience of a situation rather than an objective fact. (Note this overlaps with skills inherent to cognitive defusion, whereby flexible thinking expands opportunities for problem solving.) For example, the cognition, “My supervisor does not care about this issue” creates the perception that there may be no point to raising a concern; however, a shift in language - reflecting a corresponding shift in thought - to, “I do not *think* my supervisor cares about this issue” creates space to explore new opportunities (e.g., “Why not ask to what extent this is important to them, or where it falls in relation to other competing issues?”).

Third, we applied the speaker-listener technique when asked to facilitate or mediate a discussion between staff members and/or across workforce levels (i.e., between frontline staff and site supervisors). Specifically, we opened the floor to one speaker at a time and requested that listeners attend fully to the speaker’s message. Then, we asked listeners to summarize or reflect the speaker’s statements and provide opportunities for clarification before responding. We mirror this in our own conversations with *Champions* staff members, an intentionally parallel process wherein we model the communication skills we then ask our partners to adopt. Progress toward opening communication was reflected by several examples: the site supervisor initiated more frequent end-of-day check-ins; the supervisor also became more engaged with staff feedback; program directors engaged in discussion about frontline staff burnout. However, consistent implementation of larger components (e.g., regular meetings for leadership and frontline staff) remained challenging, and shifting leadership and staff turnover interfered with stability of improvements, though a number of staff members became more actively engaged in problem solving over time.

### Theme 3: Responding To Stress And Emergent Events Impacting Cbo Staff

*Champions* operates under difficult conditions (e.g., high burnout and a combination of workplace and personal stress) in a community facing resource scarcity, health disparities, and frequent violent crime. In particular, we became acutely aware of the cascading impact of violence and loss on our partners over time. In an especially striking three-month period in our second year of partnership, one elementary-age student died from health complications and three high school students, former volunteers for pre-k programming, lost their lives to gun violence, sending waves of grief through the community. Though studies on mental health workers, nurses, and even, to a lesser extent, scientists have examined compassion fatigue and vicarious and indirect trauma (Baird & Kracen, 2006; Hunsaker et al., 2015), it is a relatively new literature and little is known regarding prevention and intervention (Bercier & Maynard, 2015; Ledoux, 2015). Even less is known regarding their incidence and impact on youth-serving frontline staff in non-healthcare settings. However, investigators have long acknowledged the negative effect of burnout on implementation, job performance, and program quality (Damschroder et al., 2009; White, 2006). Time spent with frontline staff revealed the deeply personal impact left by loss in their community, as well as high levels of life stress and limited opportunities to engage in self-care.

#### Compassion-Based Concepts

Though compassion fatigue and vicarious trauma lack clearly-defined, evidence-based interventions (Bercier & Maynard, 2015), a growing literature suggests compassion-oriented practice may reduce negative affect (Barnard & Curry, 2011) and promote well-being and self-care (Sinclair et al., 2017). As with the broader literature on compassion, research on self-compassion - while more recent and less developed - indicates its association with individual well-being (Barnard & Curry, 2011) and emotion intelligence (Heffernan et al., 2010); improved conflict resolution, ability to compromise, and reduced self-subordination (Yarnell & Neff, 2013); and, in preliminary studies, compassionate care of patients in healthcare settings (Sinclair et al., 2017).

Similarly, recent commentaries propose that despite widespread workplace stress and suffering, systematic study of interpersonal dynamics - in particular peer response to colleagues’ stress - remains scarce. Growing evidence, however, points to the beneficial impact of peer compassion at work to improve employee mental health, enhance feelings of value and increase organizational commitment (see Dutton et al., 2017 for a review). Moreover, investigators have found that receiving, providing, or even simply observing compassionate response in the workplace relates to more positive “sensemaking” (i.e., interpretations of motives, kindness, and capacity) about colleagues, the organization, and oneself (Lilius et al., 2008). Compassionate organizing directs resource distribution toward areas of need (Dutton et al., 2017), and promotes work attitudes and performance that support the common good (Haidt, 2002 as cited in Dutton et al., 2017). Models examining compassion in organizations often extend to elements that lie outside the influence of partnered consultation (e.g., institutional structure, organizational strategy); however, the strength of evidence highlighting the potential benefits of compassion on individual employees - either as participants or bystanders of supportive interactions - lends credence to the promise of compassion as a central process in workforce capacity building.

#### Application

In addition to our efforts to encourage further compassion among the staff for each other, we adopted a broad aim to model compassionate regard toward staff and encourage them to be compassionate toward themselves. We provided crisis intervention and grief support immediately following losses or community disruptions at the request of *Champions* leadership, site supervisors, and frontline staff, and promoted self-compassion via three components: regard the self with kindness and understanding in response to struggles and perceived failure (e.g., cognitive flexibility around expectations to perform under difficult circumstances); view lived experience as part of the larger human condition (e.g., cognitive restructuring to replace a harsh or self-critical lens); and observe painful feelings and thoughts mindfully (e.g., implementation of mindfulness practices such as meditation and body scans) (Neff, 2004). We incorporated regular check-ins with individual staff members about their personal mental health, incorporating joint, non-judgmental evaluation of stress and strain, and encouraging small behavioral changes in self-care to support well-being and resilience (e.g., mindful minute, go for a walk, listen to music, engage in positive conversation not related to work). Anecdotally, our embrace of CORE principles led to deeper relationships with individual staff, stronger connections to local families, closer ties to the community, and - according to feedback from *Champions* - a perception at all workforce levels of our efforts as respectful of and responsive to individual stakeholders and the organization overall.

#### Lessons Learned

Our efforts to stay present with our partners, both through chronic organizational challenges and acute adverse events impacting the broader community, provided insight and direct exposure to the day-to-day challenges and needs of staff and the children and families they serve. Most notably, while we maintained organizational goals, identified and agreed upon together with our collaborators, we released ourselves from strict agenda-driven expectations for workshops and consultation. Though we came prepared with content that aligned with ongoing goals, we encouraged staff members to set topics based on the ebb and flow of their needs. Relatedly, we sought permission from staff members to check in around emergent and stressful life events, and personal and professional challenges, wanting to provide whatever support we could without overstepping individual boundaries. Despite limitations to time and privacy arising from the setting of our conversations, brief psychosocial support (for those interested) in the form of acknowledgement and reflective listening, paired with suggestions for strategies such as progressive muscle relaxation or meditation, facilitated deeper connections in partnership and acted as an assurance to staff at all levels that we cared about their wellbeing as individuals, not just as professional partners.

### Theme 4: Facilitating Self-Care And Peer Support Within The Research Team

Deeper connections, enriched relationships, increased time, expanded roles, and greater personal investment translated to more proximity to adverse events, and more frequent exposure to community violence and contact with grief and loss, including personal connections to adults and youth who passed away. As investigators, we hold greater access to resources, agency over responsibilities, and ability to disengage from local stressors at the end of the day - a privilege not enjoyed by *Champions* frontline staff, many of whom are local residents of the community they serve. At the same time, our presence on site during frequent lockdowns related to nearby gun violence made a gradual but significant impact on both our understanding of our partners’ lived experience, and our own mental health. Since the effects of vicarious and indirect trauma on investigators remains relatively unstudied (van der Merwe & Hunt, 2019), we turned to training, supervision, and workforce management research for insight.

#### Compassion-Based Concepts

Prior literature speaks to the importance of compassion in supervision to temper the effects of indirect trauma (Knight, 2013), and the potential of self-compassion to produce positive outcomes related to provider depression and burnout, especially as a mediator in the relationship between these outcomes and self-critical perfectionism (Richardson et al., 2018). Additionally, Beaumont & Hollins Martin (2016) propose that Compassionate Mind Training might improve student therapists’ well-being, reduce burnout and compassion fatigue, and promote resilience. Specifically, journaling, reflexivity, and group debriefing (e.g., opportunities to consider events in partnership non-judgmentally as a team, to seek support and guidance) help community-engaged researchers support ethical practice (Case, 2017; Chou & Frazier, 2019), consider consultation and partnership with objective distance, and track ongoing work to provide evidence of progress and counter self-critical thinking. Studies also have demonstrated the ability of the Gestalt two-chair technique to increase self-compassion and decrease anxiety and depression (Neff, 2004). Traditionally guided by a therapist, individuals conceptualize themselves as having two “selves” - a judgmental self and a self that receives the judgment - that then engage in a “conversation” with the goals of gradually learning to recognize the impact of listening to their self-criticism and working toward compassionately “defending themselves” in response.

#### Application

Accordingly, we (doctoral candidate Chou and faculty mentor Frazier) brought into clinical supervision a number of compassion-oriented intervention strategies (e.g., mindfulness, meditation, rhythmic breathing, cognitive defusion, compassionate self-regard). We allocated time to joint reflection and debriefing, and improving work-life balance as a means to maintaining the three “flows” of compassion: (1) outward flow (compassion *for* others); (2) inward flow (compassion *from* others); and (3) self-compassion (Gilbert, 2014 as referenced in Beaumont & Hollins Martin, 2016). Author Chou adopted journaling and reflexive practice and - with targeted learning in the functional importance and conceptual underpinnings of self-compassion - employed the Gestalt two-chair technique as a self-guided activity.

#### Lessons Learned

Broadly speaking, we are grateful for our rich collaboration and deep connections with the *Champions* team; however, we were, in truth, unprepared for the potential impact of increased personal relevance and exposure to local incidents such as gun violence and loss. Moving forward, we plan to implement a standard practice of journaling and both individual reflexivity and group debriefing at the start of a partnership. Further, we aim to incorporate compassion-based literatures with particular focus to self-compassion as a preventive measure and to build resilience among community-engaged researchers. Lastly, we will continue incorporating conversations about work-life balance, both as a general topic of professional development and as a regular check-in to course correct as needed, within our research team and with trusted professionals who might bring objective support and fresh perspective.

## Discussion

Compassion-Oriented Reflection and Engagement evolved as a direct response to individual, organizational, and community-wide challenges; it framed our experience and advanced a more holistic integration of empathy and science. In this way, CORE has informed our practice to promote wellness in *Champions*’ personal and organizational functioning and, perhaps most importantly, revealed opportunities to extend our efforts as academic collaborators beyond conventional transport of traditional evidence-based intervention packages. Our role and function exceeded the original expectations of our partnered work and, as a result, we expanded our practice beyond traditional prospective research methodology often found in DIS science. While we continued to inform each step through quantitative and qualitative means (e.g., group discussions with staff, questionnaires and surveys on burnout, job resources and control) and have worked to maintain a rigorous retrospective on our activities through detailed field notes and debriefing discussions, the introduction of CORE components to our work has resulted in considerable growth. Robust literatures speak to the high potential for compassion-based content as a lever for change to enhance the experience and impact of community-engaged DIS science, and future work is poised to examine CORE as a driver of consultation and potential mechanism for community-wide change.

Though systematic infusion of compassion-oriented literature into our work came later in our partnership with *Champions*, its values inherently align with long-standing efforts of our team. Just as we have called for a redefinition of traditional research concepts like feasibility (Frazier et al., 2008) and sustainability (Frazier et al., 2019), historically we have placed strong emphasis on the vision, perspectives, and lived experience of partnering community stakeholders. Consultative decisions lean heavily toward highlighting *Champions*’ existing strengths and addressing needs and barriers that partners identified as most urgent, rather than pushing forward adhering to original, even collaboratively determined, implementation goals. Our efforts to operate flexibly come from our desire to bridge our own mental health and youth services expertise with our partners’ knowledge and proficiency in youth programming and community engagement that carries equal - if not greater - weight in driving collaboration. CORE aligns well with these aspirations, as it directs consultation toward acknowledgement, empathy, empowerment and action around local strengths, challenges, and perspectives.

In many ways, CORE has stretched us beyond our expertise and led our work to areas of highest priority for our partners, allowing us to search for, translate, and mobilize science that bears most directly on their expressed needs. Reliance and focus on CORE has allowed us to suggest and model self-care practice that we hope will generate sustainable and lasting change for *individual* staff. While conceptualizing sustainability in this way may differ from traditional notions of *organizational* change, research documenting high turnover in CBOs with transient, pre-professional staff (Frazier et al., 2019) indicates potential for individual professional and personal development to have even greater influence than strategies targeting organizational structure and procedure. As teachers, student supporters, and other full- and part-time frontline providers, aids, coaches, advocates and instructors cycle through employment with community-based organizations, consultation that builds their individual capacities *with their transience in mind* may support a larger public health goal of reducing stigma and disseminating scientific knowledge and impact. Hence, focusing on individuals’ personal and professional development instead of change to organizational culture and structure may capitalize on the transient nature of community workforces, making predictable turnover more of an opportunity than an obstacle in community-engaged implementation science.

Perhaps most notably, our conversations with *Champions* team members across hierarchical levels have revealed a qualitative appreciation for the longevity and depth of our partnership. Time and again, partnered staff voiced feedback that converged around the strength of our having arrived on site, observed a vast array of challenges, and “rolled up our sleeves.” Frontline staff in particular have noted the difference between our willingness to stay present in their work and the impressions left by previous collaborators who have arrived with strict agendas, initiated and adhered rigidly to their planned work, and discontinued partnership if resources were too few, challenges too great, enthusiasm too limited, or goals and priorities too misaligned. Though we recognize a need to conduct more rigorous empirical examination of causal effects and mechanisms, we believe CORE principles and processes have allowed for the development of a rich and responsive partnership, built on a foundation of mutual trust and respect, and offering ongoing lessons in DIS science of “what matters, when” in the transport of evidence-based practices (Schoenwald & Hoagwood, 2001).
